# Intrusion of posterior teeth using miniplates: intrusive mechanics is not the same as intrusion force

**DOI:** 10.1590/2177-6709.26.5.e21ins5

**Published:** 2021-10-25

**Authors:** Suzana T. M. P. M. APOLINÁRIO, Aparecida Fernanda MELOTI, Ertty SILVA, Mauricio de Almeida CARDOSO, Alberto CONSOLARO

**Affiliations:** 1 Faculdade São Leopoldo Mandic, Programa de Pós-Graduação em Ortodontia (Campinas/SP, Brazil).; 2 Universidade de São Paulo, Faculdade de Odontologia de Bauru (Bauru/SP, Brazil). Universidade de São Paulo, Faculdade de Odontologia de Ribeirão Preto, Programa de Pós-Graduação em Odontopediatria (Ribeirão Preto/SP, Brazil).; Universidade de São Paulo, Universidade de São Paulo, Faculdade de Odontologia de Ribeirão Preto, Programa de Pós-Graduação em Odontopediatria, Ribeirão Preto, SP, Brazil

**Keywords:** Intrusion, Skeletal open bite, Root resorption, Intrusive mechanics, Miniplates, Absolute anchorage

## Abstract

**Objective:**

Biologically explain some of the bone mechanisms involved in the intrusion, or intrusive effect, of teeth submitted to skeletal open bite correction using four miniplates.

**Methods:**

The results of dental intrusion were measured and compared in 3D reconstructions of cone beam computed tomography scans taken before and after treatment of 20 patients with skeletal open bite, aged between 18 and 59 years.

**Results:**

The results allow deducing that the compression and traction forces biologically promoted deformation or deflection of the osteocyte network that controls bone design, and these effects involved the external and internal surfaces of the bone, with the formation of new layers, including the cervical portion of the alveolar bone crest. This helps understanding how dental intrusion occurs in intrusive mechanics, whose forces are of inclination rather than intrusion. The root resorptions caused by the use of miniplates were insignificant, due to the more homogeneous distribution of forces in the several teeth simultaneously involved.

**Conclusion:**

Imaging studies in CT scans tend to capture in details the subperiosteal and endosteal phenomena of dental intrusion - before and after the application of intrusive mechanics -, in the form of a set of modifications called dental intrusion or intrusive effect .

## RESUMO

**Objetivo:**

Explicar, biologicamente, alguns dos mecanismos ósseos envolvidos na intrusão, ou efeito intrusivo, de dentes submetidos à correção da mordida aberta esquelética por meio do uso de quatro miniplacas.

**Métodos:**

Foram mensurados, em reconstruções 3D de tomografias computadorizadas de feixe cônico, os resultados da intrusão dentária, comparando-se o antes e o depois em 20 pacientes com mordida aberta esquelética, com idades entre 18 e 59 anos.

**Resultados:**

Os resultados permitem deduzir que as forças de compressão e de tração promovem, biologicamente, deformação ou deflexão da rede osteocítica controladora do *design* ósseo, e esses efeitos envolvem as superfícies externas e internas do osso, com formação de novas camadas, incluindo a parte cervical da crista óssea alveolar. Isso ajuda a compreender como ocorre a intrusão dentária nas mecânicas intrusivas cujas forças são de inclinação, e não de intrusão. As reabsorções radiculares promovidas pelo uso de miniplacas são insignificantes, em função da distribuição mais homogênea das forças nos vários dentes simultaneamente envolvidos.

**Conclusão:**

Os estudos imagiológicos tendem a captar, nas tomografias, cada vez mais detalhadamente os fenômenos subperiosteais e endosteais da intrusão dentária nos pacientes - antes e depois da aplicação das mecânicas intrusivas -, na forma de um conjunto de modificações que se chama intrusão dentária ou efeito intrusivo.

## INTRODUCTION

Teeth are repositioned in the bone thanks to bone remodeling, which represents a set of events in the periodontium and jaw bones that allow the desired reshaping of bone.^1^ Dental movement alone does not justify the esthetic and functional benefits promoted by orthodontics, since orthodontic treatment is the result of an induced movement of teeth and bones. Previously, it was thought that tooth movement was done through the bone, but advances in knowledge have revealed that teeth and bone continually reposition and harmoniously reshape themselves together.

**Figure 1:**
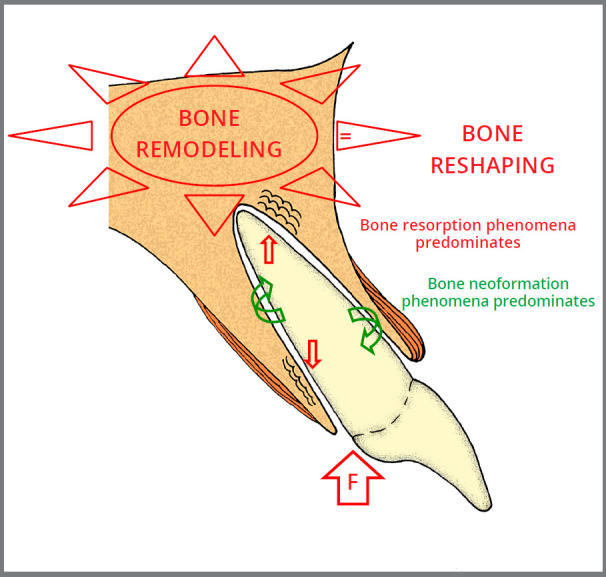
Anterior teeth: diagram representing the predominant phenomena in each alveolar bone area under the action of intrusive mechanics, which promotes bone reshaping based on directed remodeling of the bone, with inclination forces for tooth intrusion to occur. Red arrows = compression areas. Green arrows = areas of stretch or tension. F = Force.

In clinical practice, pure and specific intrusion forces parallel to the long axis of the root, and perpendicular to the alveolus bottom are not used.^2^ Intrusive mechanics can have the effect of moving teeth deeper into the jaws bones, but the forces that promote these effects are not those of intrusion, but rather of inclination^2^ (Figs 1 e 2). The position of teeth in the alveolar processes and the root inclinations in relation to the dental crown in single and multi-rooted teeth reinforce the reasoning that in orthodontic clinical practice, there are no pure intrusion forces.^2^

**Figure 2:**
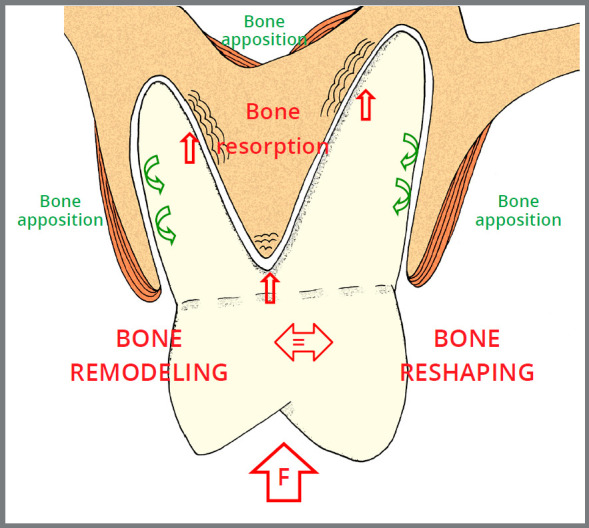
Posterior teeth: diagram representing the predominant phenomena in each alveolar bone area under the action of intrusive mechanics, which promotes bone reshaping based on directed remodeling of the bone, with inclination forces for tooth intrusion to occur. The maxillary sinus is pointed out. Red arrows = compression areas. Green arrows = areas of stretch or tension. F = Force.

**Figure 3:**
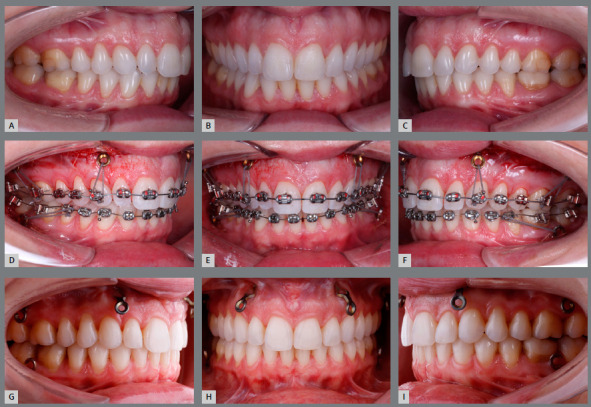
A 37-year-old patient whose teeth were submitted to intrusive mechanics with miniplates, and the result after a year. **A, B,** C) Before treatment; **D, E,** F) after brackets bonding and miniplates activation; **G, H,** I) treatment completed.

It is noteworthy that:

1. *Intrusion force:* acts on the tooth’s longitudinal axis or parallel to it, on the root structure, forming right angles with the tangent that passes parallel to the bottom of the alveolus.
2. *Intrusive effect, or intrusion*: it is the displacement of the tooth to a more intraosseous position than the existent position (Fig 3). This intrusion can be achieved in several ways, with different forces and mechanisms - which, in conjunction, can be called intrusive mechanics^2^. Perhaps the least effective force for this purpose is pure intrusion.

### THE BIOLOGICAL FOUNDATIONS OF INTRUSIVE MECHANICS

The effective shock absorber represented by the periodontal ligament and the extracellular matrix, with collagen fiber bundles, is prepared to receive and dissipate forces applied parallel to the long axis of the root. The sustaining periodontium absorbs intrusive forces even if they are intense and complex, such as masticatory forces. Masticatory loads, as intense as they may be, do not compress the periodontal ligament at the dental apex, do not damage the vascular bundle that enters the dental pulp, nor promote its necrosis.

Even when experimentally applied in animals, intrusive forces are still inclination forces, as shown in Figures 1 and 2.

The inclination of the roots, from the point of their cervical emergence, causes the experimental pure forces of intrusion at angles perpendicular to the occlusal surface of the molars, to exert inclination forces on their roots. Even if the professional exerts apparently intrusive forces, they indeed are not, due to the inclination of the teeth and their roots - that is, there are no pure intrusion forces in tooth movement. In experimental studies,^3^ ^-^ ^6^ resorptions have been microscopically observed in the apical region and in the root surfaces facing the bifurcations.

The intrusive effect of inclination forces during the mechanics known as intrusive (Fig 3) can, in part, be explained by regional orthopedic stimulus to reshaping, represented by the forces on the bone, at the expense of the periosteal and endosteal tissues.^1^ The periosteum reacts to stimuli or aggressions of low intensity and long duration with the formation of new bone layers on the cortical surfaces; that is, at the periosteum-cortical interface.^1^ ^,^ ^7^ ^,^ ^8^ This reaction capacity can modify the shape of the bone in question, by increasing its volume or thickness. The endosteal and periosteal surfaces of the bone are compensated by the cellular information transferred from the periodontium to the periosteum by the osteocyte network controlling the bone design.^1^ ^,^ ^7^ ^,^ ^8^

Force-induced bone deformation, or deflection, modifies the final maxillary shape, as remodeling allows the tissue to meet the new functional demands transmitted to the entire bone via the osteocyte network^1^. Subperiosteal cortical resorptions and appositions can occur on the external part of alveolar processes, where the teeth are being intruded (Figs 1 and 2), which can also occur on internal surfaces, such as the walls of the maxillary sinus and of the nasal cavity. This same phenomenon on endosteal surfaces changes and rearranges the bony trabeculae, both in terms of their spatial distribution and the thickness and length of the bony trabeculae.^1^

In intrusive mechanics, the tooth that entered into the bone space and was repositioned therein, also called intruded tooth, is repositioned in relation to the bone as a whole, and in relation to the other teeth, by the application of stimuli of an orthodontic and orthopedic nature (Fig 3). The height and shape of periodontal tissues can be altered without modifying the biological distances of periodontal tissues and without affecting the biological viability of the pulp tissues.

Intrusive mechanics promotes a spatial rearrangement of the bone in relation to the tooth - by means of phenomena known as orthopedics - and, simultaneously, the tooth repositions itself orthodontically, upon inclination forces. As a result of this synergy, the tooth is in a new position in relation to the bone and other teeth in the dental arch (Fig 3). 

### TOOTH RESORPTION AND INTRUSIVE MECHANICS

The high risk of root resorption^3^ is always mentioned when it comes to intrusion movements, which are mentioned when referring to intrusive mechanics - intrusive mechanics does not mean intrusion force, as we explained before. There are many studies that have revealed that there is no such relationship between tooth resorption and intrusion force^5^ ^,^ ^9^ ^-^ ^13^ The forces that become most concentrated and potentiated in points of the periodontal ligament, with death of cementoblasts, are those of inclination, which characterize the intrusive mechanics.^7^

The use of specific appliances, with absolute anchorage by means of plates and osseointegrated implants in dogs’ teeth,^3^ induced imagiologically insignificant root resorption, despite the intrusive effects obtained. The same occurred microscopically after a period lasting between four to seven months.^4^ ^,^ ^5^ ^,^ ^6^ Clinical trials with important intrusive effects have also revealed a very low or non-existent rate of tooth resorption.^14^ ^,^ ^15^ Appliances with absolute anchorage tend to distribute more homogeneously the forces that are applied, dissipating and eliminating the points of concentration of forces in periodontal tissues. This reduces the possibility of death of cementoblasts along the root, and reduces the chance of root resorptions.^16^

Pure intrusive forces in Orthodontics exist only experimentally and, when resorptions occur at the apexes and bifurcations of these teeth, they are detectable only under a microscope, as they cannot be diagnosed by imaging exams.^3^ ^-^ ^6^ Studies have revealed that when intrusive forces are eliminated, neighboring tissues quickly repair the resorbed areas.

### DEMONSTRATIVE EXPERIMENT OF INTRUSIVE EFFECTS

In a research project conducted for a master’s dissertation, Apolinário^17^ used temporary skeletal anchorage to quantify dental intrusion into the bone in patients with skeletal open bite, using miniplates, as previously suggested.^18^ ^,^ ^19^

Miniplates are more effective alternatives than mini-implants, as they bear loads of greater intensity and to a greater extent of the dental arches.^20^ Consolaro^21^ mentioned that skeletal anchorage is necessary for bone remodeling to redefine morphology, esthetics and function in cases of deformities, always supported by orthodontic appliances. 

Molar intrusion is difficult to achieve with conventional orthodontics - in these cases, orthognathic surgery is indicated. However, miniplates in the zygomatic bone region represent an alternative mechanics,^5^ as they are predictable, having a success rate of 98.6%.^22^

In Apolinário’s study,^17^ intrusion of posterior teeth, necessary for the correction of the anterior open bite, was distributed among the four hemiarches, with the aid of four miniplates used as anchorage. The sample consisted of 40 DICOM files (20 before and 20 after) from cone beam computed tomography scans requested for 3D evaluation before and after orthodontic treatments performed with the aid of miniplates, between the years of 2014 and 2018. The patients were of both genders, aged between 18 to 59 years, with anterior open bite, with overbite of up to -7.6 mm, orthodontically treated with maxillary and mandibular posterior intrusion, anchored on four miniplates; whose tomographs had a FOV (field of view) greater than or equal to 23 x 17 cm. In all cases, intrusion of maxillary and mandibular posterior teeth was obtained. 

The distances from the cementoenamel junction in relation to the axial plane were measured in the central incisors, maxillary first and second molars; and so were the distances between the apex of the incisors and the mesial root of the maxillary first and second molars to the axial plane, in the pre- and post-treatment phases. In the mandibular teeth, the distances from the cementoenamel junction to the mandibular plane were measured in the central incisors and mandibular first and second molars; and so were the distances between the mandibular plane and the apex of the incisors and the mesial root of the mandibular first and second molars, in the pre- and post-treatment phases.

The values of the measurements before and after the intrusion were found to be statistically significant for the maxillary first molars on the right and left sides in relation to the axial plane, and for the mandibular first and second molars on the right and left sides in relation to the mandibular plane. In this study, it could be concluded that the amount of dental intrusion obtained in both arches was similar to the values found in the literature; however, with the use of four posterior miniplates, the intrusion was enhanced in both the maxillary and mandibular arches, when compared with the intrusion performed in a single arch. The use of miniplates to obtain the intrusion of teeth into the jaws bones was carried out without root resorption or, when root resorptions were identified in the images, they were so small that their extension was questionable.

## FINAL CONSIDERATIONS

The natural inclination of the roots favors the tooth movements in intrusive mechanics to be of inclination type. There are compression forces in some areas of the periodontal ligament, with deformation or deflection of the osteocyte network that controls bone design. In other areas, there is deformation of the osteocyte network, due to tension forces. These effects (Fig 3) involve the external and internal surfaces of the bone in the alveolar process, with the formation of new layers and areas of resorption, including the cervical portion of the alveolar bone crest.

In intrusive mechanics with the use of miniplates, alveolar remodeling of an orthodontic nature occurs, associated with modification of the internal and external bone structure, to meet the demands of forces with orthopedic nature.^1^ The intrusive effect in so-called intrusive mechanics may be the result of alveolar remodeling induced by inclination forces and changes in bone volume resulting from subperiosteal bone formation in the external part of the alveolar process^1^ ^,^ ^5^ ^,^ ^8^ The more homogeneous distribution of forces with absolute anchorage on miniplates explains the insignificant level of root resorption.^16^

Refined imaging studies, such as the one reported by Apolinário,^17^ capture these subperiosteal and endosteal phenomena in high precision computed tomographs taken before and after the application of intrusive mechanics; that is to say, these phenomena represent the final effect called dental intrusion, or intrusive effect.
